# Distinct effect of partial sleep deprivation associated with gray matter changes in young and old adults

**DOI:** 10.3389/fnagi.2025.1640653

**Published:** 2025-08-05

**Authors:** Xiao Fu, Yanni Shi, Hui Xu, Dongwu Xu

**Affiliations:** School of Mental Health, Wenzhou Medical University, Wenzhou, China

**Keywords:** sleep deprivation, young adults, old adults, cortical thickness, cortical surface area

## Abstract

**Background:**

Sleep deprivation (SD) exerts adverse effects on human brain. However, whether there were distinct effects of partial SD associated with gray matter changes in young and old adults, respectively, remains unclear.

**Methods:**

42 young adults and 38 old adults were enrolled in this study. All participants underwent MRI scanning, and FreeSurfer 5.3 was used to calculate cortical thickness (CT) and cortical surface area (CSA). Paired two samples *t*-tests was conducted to explore CT and CSA changes. Partial SD involved restricting total sleep time to approximately three hours, compared with baseline sleep conditions.

**Results:**

Young adults exhibited increased biopsychological response (Sickness-Q score) following partial SD compared to the rested condition, and similar trend was observed in old adults in SD. Young adults exhibited decreased CSA of left caudal middle frontal cortex and CT of entorhinal cortex (EC), but increased CSA of left temporal pole and CT of right insula after SD. However, old adults showed increased CSA and CT in widespread brain regions, including left superior frontal cortex, left isthmus cingulate cortex and right EC. While young adults showed a significant positive correlation between percent change of CSA of left EC and the biopsychological response, old adults showed a significant negative correlation between percentage change in CT of the left isthmus cingulate cortex and biopsychological response.

**Conclusion:**

Distinct effect of partial SD associated with gray matter changes were observed in young and old adults, respectively. These findings shed light on SD might affect brain structures differently in young adults and old adults.

## 1 Introduction

Sleep is a complex physiological state, during which humans spend approximately one-third of their lives (Kendzerska et al., 2014). It is crucial for maintaining physical and mental wellbeing (Foster, 2020; Matricciani et al., 2019; Tempesta et al., 2018). However, a large portion of the population suffers from insufficient sleep and various sleep disorders, including excessive daytime sleepiness, insomnia, abnormal movements or behavior during sleep and inability to sleep at the desired time (Chokroverty, 2010). Common forms of sleep loss are total SD, sleep restriction (partial SD) and sleep fragmentation or disruption (Reynolds and Banks, 2010). Sleep restriction is a reduction in sleep time below an individual’s usual baseline or the amount of sleep needed on a regular basis to maintain optimal performance. This is probably the most frequently experienced form of sleep loss in everyday life (Banks and Dinges, 2007). Furthermore, the aging process disrupts the physiology of sleep and reduces total sleep time (De Crescenzo et al., 2022). Increasing evidence links sleep to many health problems, such as hypertension, obesity, cardiovascular diseases, metabolic diseases, and diminished quality of life. In addition, sleep disturbances have been linked to impaired cognitive function and increased likelihood of occupational errors (Cappuccio and Miller, 2017; Hale et al., 2020; Medic et al., 2017; Ohayon et al., 2002).

Experimental studies on the effects of sleep loss in humans date back to the late 19th century, with early reports investigating the consequences of acute sleep deprivation on cognitive and physiological functions (Patrick and Gilbert, 1896). Prolonged sleep deprivation (e.g., 90 h) was shown early on to impair memory and reaction time, laying the groundwork for extensive later research. Total sleep deprivation remains the most common experimental approach in sleep studies (Reynolds and Banks, 2010). In real-world settings, total sleep deprivation is uncommon. Instead, partial sleep deprivation—sleeping less than one’s habitual or required amount—is far more prevalent. Studies show that even limiting sleep to five hours per night can significantly impair simulated driving performance (Banks et al., 2004).

Experimental models of SD have revealed significant impacts on brain structure and function. It has found that SD is associated with increased cerebral blood flow, decreased metabolic rate, and markers of neuronal synaptic activity in several brain regions (Elvsåshagen et al., 2019; Thomas et al., 2000). Furthermore, paired comparisons between the two morning scans for the deprivation group showed a significant thinning primarily in the bilateral medial parietal cortices, including the precuneus and posterior cingulate cortices, after 23 h of SD (Elvsåshagen et al., 2017). Additionally, over six hours of SD can induce changes in neuronal activity across multiple brain regions, including the lateral hypothalamus, paraventricular nucleus, arcuate nucleus, and body. These adverse effects can last up to 48 h (Fifel et al., 2018).

Notably, the studies mentioned above primarily involved young adult participants, with mean ages centered around 22 years. This consistent focus on young adults provides a foundation for examining how SD uniquely affects cognitive and neural functioning in this developmental stage. After SD, young individuals show reduced inhibitory control and increased impulsivity when performing inhibitory control tasks, especially in the presence of negative stimuli (Shao et al., 2013; Shao et al., 2014). Acute SD can induce changes in gray matter structure of the right frontal pole, right superior frontal gyrus, and right middle frontal gyrus, while there was a significant decrease in gray matter volume and CT in the right temporal pole (Sun et al., 2020). SD results in more severe cognitive impairment in young people compared to older adults.

The brain functional and structural changes associated with SD are age-related. One study using a verbal encoding task found high sleep-related activation in the anterior hippocampus in older adults and low activation in young adults (Jonelis et al., 2012). Functional and structural magnetic resonance imaging (MRI) studies have found that only in younger individuals, resting-state functional connectivity and gray matter volume in the anterior insula change after SD (Long and Cheng, 2019; Long et al., 2020). Only younger adults show a correlation between the connectivity of the medial temporal lobe network and sleep quality (Liu et al., 2018). These findings may suggest that young and older adults show different brain changes underlying SD. However, whether there were distinct effects of SD associated with gray matter changes in young and old adults, respectively, remains unclear. To address this question, we extended findings from previous experimental partial SD studies—including Long et al. (2020)—by introducing key methodological improvements. Unlike previous studies using voxel-based morphometry (VBM), we employed surface-based morphometry (SBM), which is more sensitive to subtle cortical changes. We also integrated behavioral assessments and considered a broader set of covariates, enhancing both the interpretability and generalizability of our findings.

SD has been shown to induce inflammatory responses, (Chennaoui et al., 2011; Everson, 2005; Zielinski et al., 2014) which can lead to brain structural alterations (Marsland et al., 2015). Brain structural changes may reflect an individual’s biopsychological response to immune activation, encompassing both physiological and psychological dimensions (Kraft and Kraft, 2021). The Sickness-Q questionnaire has been proved to be a validated tool to assess perceived sickness behavior in humans, focusing on symptoms like fatigue, mood disturbances, and bodily pain resulting from inflammatory activation (Andreasson et al., 2018). It captures the subjective experience of individuals under conditions of systemic inflammation, making it a useful measure for understanding the broader impact of immune activation on health and wellbeing (Lasselin et al., 2018). While there are indications of potential associations between inflammation, brain structural changes, and subjective sickness symptoms, it remains to be explored how SD affects both brain structure and biopsychological responses across different age groups. In this study, we aim to investigate these relationships further.

To address this gap, we employed surface-based morphometry to examine the distinct effects of SD on CT and CSA in young and older adults. We hypothesized that:

(1) Compared to normal sleep conditions, young adults would show decreases in CT and CSA primarily in frontal regions (e.g., caudal middle frontal cortex);
(2) Older adults, in contrast, would exhibit increases in CT and CSA mainly in temporal regions (e.g., superior temporal gyrus and EC) following partial SD;
(3) These structural changes would be significantly correlated with biopsychological responses, but the direction of the associations would differ between age groups.

## 2 Materials and methods

### 2.1 Participants

This study utilized data from the Stockholm Sleepy Brain Project, publicly available at OpenNEURO.^1^ The Stockholm Sleepy Brain Study aimed to investigate the overall effects of sleepiness on brain structure and function, with a particular focus on emotional processing. Using functional and structural magnetic resonance imaging, the study examined changes in resting-state brain connectivity and neural responses during experimental tasks involving emotional mimicry, empathy for others’ pain, and cognitive reappraisal of emotional stimuli as well as the brain structural changes before and after sleep deprivation. Participants were included if they were aged 20–30 or 65–75, met MRI safety requirements, had normal or correctable vision using contact lenses, and were right-handed. Exclusion criteria included a history of psychiatric or neurological disorders, hypertension, diabetes, use of psychoactive or immune-modulating drugs, daily nicotine consumption, and excessive caffeine intake. Initial screening used tools including the insomnia severity index (ISI), (Bastien et al., 2001) hospital anxiety and depression scale (HADS), (Zigmond and Snaith, 1983) and Karolinska Sleep Questionnaire (KSQ), (Kecklund and Åkerstedt, 1992) Epworth Sleepiness Scale (ESS), (Johns, 1991) and Toronto Alexithymia Scale-20 (TAS-20), (Bagby et al., 1994) to exclude participants with insomnia, anxiety, depression, irregular sleep patterns, or snoring/apnea issues. Elderly participants also completed a Mini Mental Test (MMT) (Folstein et al., 1975) at their first visit to ensure no significant cognitive deficits. A total of 86 healthy adult participants were recruited for this study. After excluding five subjects due to missing T1 images before or after partial SD and one subject due to artifacts, 80 participants remained. Detailed participant information is provided in Table 1. Each participant underwent two MRI scans: one after a full night’s sleep (referred to as SA, sleep adequate) and another after partial SD, with the scans conducted one month apart. Here, “SA” denotes the baseline sleep condition, where participants experienced a full night of adequate sleep prior to the scan, serving as the reference point for comparing the effects of sleep deprivation. The scans were scheduled in the afternoon or evening. Prior to each MRI scan, participants completed several self-report questionnaires to assess their current health status and psychological state. These included the Self-Rated-Health (SRH5) (Eriksson et al., 2001), Positive and Negative Affect Schedule (PANAS-positive and PANAS-negative) (Watson et al., 1988), and Sickness Questionnaire (Sickness-Q) (Andreasson et al., 2018). To ensure accurate sleep monitoring, participants used sleep recording devices throughout the night. Sleep recording was conducted using portable Embla and Vita port systems in the homes of the participants. These solid-state, portable sleep recorders utilized a standard electrode montage with silver/silver chloride electrodes for EEG sleep recording. Specifically, EEG electrodes were placed at C3 and C4 positions, referenced to the contralateral mastoid. Two submental electrodes were used for electromyography, and one electrode was placed at each outer canthus of the eyes for electrooculography. Data analysis was performed according to the American Academy of Sleep Medicine criteria, including detailed sleep staging and calculation of wake time as a percentage of total sleep time. For the SD condition, participants were required to go to bed 3 h before their usual wake-up time and were forcibly awakened after 3 h of sleep. To minimize order effects, participants were randomly assigned to either start with the SD condition followed by the SA condition or vice versa. All participants provided written informed consent prior to participation. The data used in this study was obtained from the publicly available Stockholm Sleepy Brain Study, originally conducted in Sweden and approved by the Regional Ethics Review Board of Stockholm. Our analysis constitutes a secondary use of anonymized data, which carried out at Wenzhou Medical University, where ethical approval for the current analyses was obtained. The study was conducted in accordance with the Declaration of Helsinki.

**TABLE 1:** Comparison of demographic and psychometric variables between older and younger groups.

Variables	Total (*n* = 86)	Old (*n* = 39)	Young (*n* = 47)	Statistic	*P*
BMI, mean ± SD	23.69 ± 3.36	24.68 ± 3.42	22.87 ± 3.10	*t* = 2.56	0.012
HADS anxiety, M (Q_1_, Q_3_)	2.00 (1.00, 3.00)	1.00 (0.00, 2.00)	3.00 (1.00, 4.00)	*Z* = −2.87	0.004
HADS depression, M (Q_1_, Q_3_)	1.00 (0.00, 2.00)	1.00 (0.00, 2.00)	1.00 (0.00, 1.00)	*Z* = −0.71	0.478
ISI, M (Q_1_, Q_3_)	10.00 (8.00, 11.00)	9.00 (8.00, 10.50)	11.00 (9.00, 12.00)	*Z* = −2.92	0.004
KSQ Sleep Quality Index, M (Q_1_, Q_3_)	5.25 (5.00, 5.50)	5.25 (4.88, 5.62)	5.25 (5.00, 5.50)	*Z* = −0.57	0.571
KSQ Wakeup Symptom Index, M (Q_1_, Q_3_)	5.33 (4.67, 5.67)	5.67 (5.33, 5.67)	5.00 (4.33, 5.33)	*Z* = −5.27	< 0.001
KSQ Snoring Symptom Index, M (Q_1_, Q_3_)	6.00 (5.67, 6.00)	5.67 (5.33, 6.00)	6.00 (6.00, 6.00)	*Z* = −4.31	< 0.001
KSQ Sleep Symptom Index, M (Q_1_, Q_3_)	5.40 (5.00, 5.80)	5.40 (5.20, 5.70)	5.40 (5.00, 5.80)	*Z* = −0.67	0.503
TAS20 total, M (Q_1_, Q_3_)	50.00 (46.00, 55.00)	48.00 (45.50, 58.00)	51.00 (46.50, 54.50)	*Z* = −0.58	0.560
ESS, M (Q_1_, Q_3_)	8.00 (5.00, 10.00)	8.00 (6.00, 11.00)	7.00 (5.00, 9.00)	*Z* = −1.79	0.073
Sex (%)				χ^2^ = 0.00	0.984
Female	44 (51.16)	20 (51.28)	24 (51.06)		
Male	42 (48.84)	19 (48.72)	23 (48.94)		

t: *t*-test; *Z*: Mann–Whitney test; χ^2^: chi-square test; -: Fisher exact. SD, standard deviation; M, median; Q_1_, 1st quartile; Q_3_, 3st quartile; BMI, body mass index; HADS anxiety, hospital anxiety and depression scale–anxiety subscale; HADS, depression: hospital anxiety and depression scale–depression subscale; ISI, insomnia severity index; KSQ, Karolinska Sleep Questionnaire; TAS20 total, Toronto Alexithymia Scale; ESS, Epworth Sleepiness Scale.

### 2.2 MRI data acquisition and preprocessing

In the Stockholm Sleepy Brain Project, T1-weighted structural images were acquired using a General Electric Discovery 3T MRI scanner. High-resolution structural images were acquired with the following parameters: echo time = 30 ms, repetition time = 2.5 s, flip angle = 75°, slice thickness = 1 mm, field of view = 240 mm, matrix size not explicitly stated (original text does not provide a specific value for matrix size), voxel size = 1 mm^3^ × 1 mm^3^ × 1 mm^3^, and it covered the whole head in sagittal orientation.

Data reconstruction and preprocessing were performed using a modified version of the FreeSurfer pipeline within the FreeSurfer Image Analysis Suite version 5.3.^2^ Detailed information on acquisition parameters, reconstruction, and preprocessing of the MRI data, were described in previous studies (Onoda and Yamaguchi, 2013). Briefly, the FreeSurfer processing stream involved several steps, including removal of non-brain tissue, Talairach transformation, intensity normalization, tessellation of gray/white matter boundaries, topology correction, surface deformation, registration to a common spherical atlas, and cortical surface reconstruction. All structural images were reviewed by a technician immediately after acquisition to ensure that scans did not have significant issues such as artifacts or substantial movement. Detailed explanations of the quality control procedures for the Stockholm Sleepy Brain Project can be found in relevant literature.^3^

### 2.3 Statistical analysis

The demographic and clinical characteristics of all participants were analyzed using SPSS software, version 25.0. While chi-square tests were used to examine gender difference, independent samples *t*-tests were used to evaluate group differences in other demographic variables and clinical characteristics.

For structural gray matter difference, *t*-test was conducted to examine group differences in CSA and CT. The significance threshold was set at *P* < 0.05 after false discovery rate (FDR) correction for multiple comparisons across each MRI modality.

Finally, in order to explore whether structural gray matter changes associated with clinical characteristic, Pearson correlation analysis was done for significant structural gray matter changes after SD. The significance threshold was set at *P* < 0.05.

## 3 Results

### 3.1 Participants and characteristics

During the baseline condition, the average total sleep time for the younger group was 8.51 ± 0.82 h, while for the older group it was 8.47 ± 0.76 h. Under conditions of sleep restriction, the younger participants obtained an average of 2.89 ± 0.58 h of sleep, whereas the older participants had an average of 2.51 ± 0.38 h. Old adults showed higher body mass index than young adults (*t* = 2.56, *P* = 0.012), while lower anxiety level (assed by HADS Anxiety scale) was observed in old adults related to young adults (*Z* = −2.87, *P* = 0.004). Older adults had significantly lower ISI scores (*Z* = −2.92, *P* = 0.004), but higher scores on the KSQ Wakeup Symptom Index (*Z* = −5.27, *P* < 0.001) and KSQ Snoring Symptom Index (*Z* = −4.31, *P* < 0.001) compared to younger adults. There were no significant group differences in KSQ Sleep Quality Index (*Z* = −0.57, *P* = 0.571), the KSQ Sleep Symptom Index (*Z* = −0.67, *P* = 0.503), TAS-20 total scores (*Z* = −0.58, *P* = 0.560), ESS scores (*Z* = −1.79, *P* = 0.073) and HADS Depression scores (*Z* = −0.71, *P* = 0.478). Detailed information on these variables is presented in Table 1.

**FIGURE 1:**
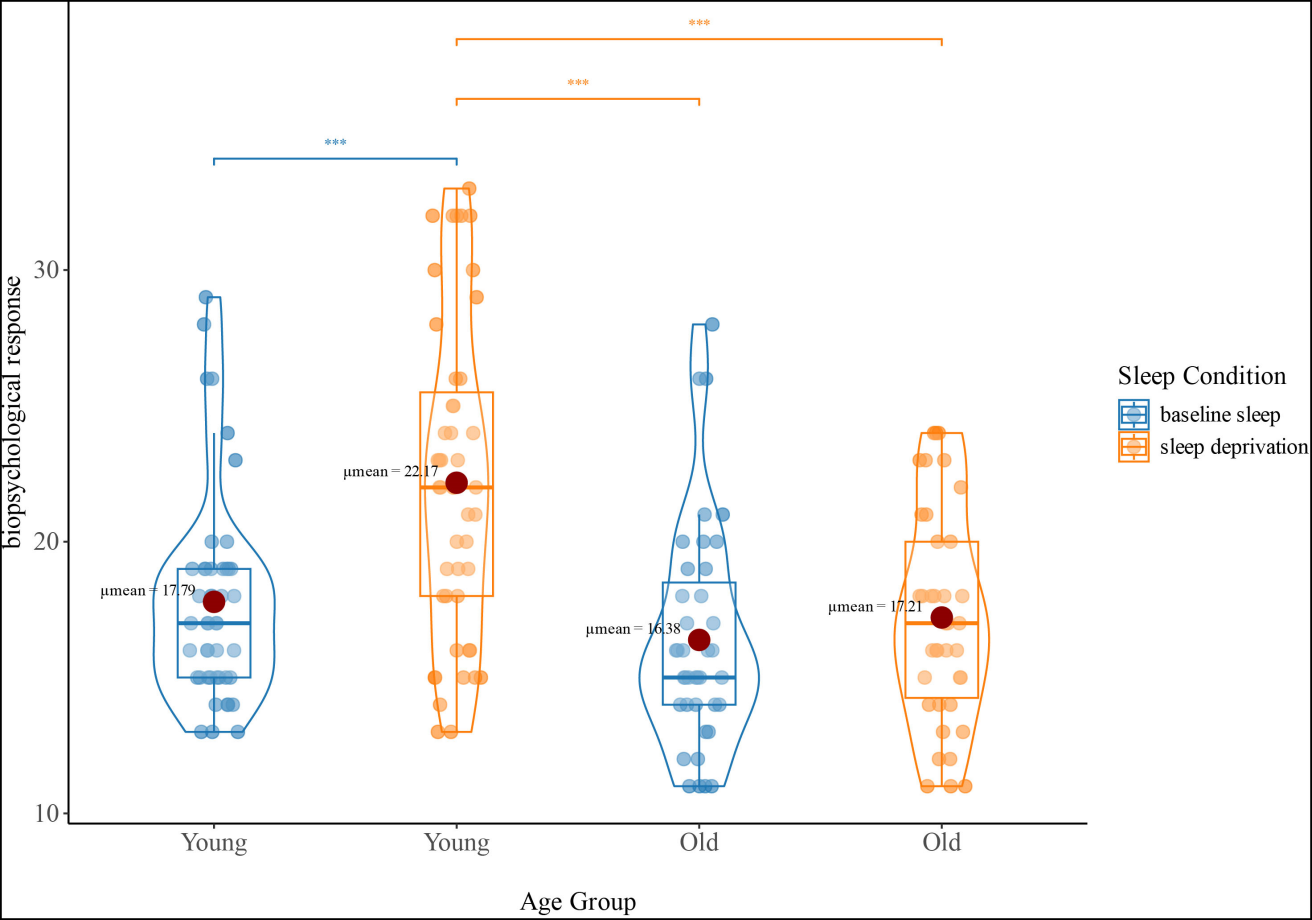
Interaction effect (age group-by-sleep condition) of biopsychological response. The blue violin plots represent the biopsychological response (reflecting sickness-Q score) of the young and old groups under baseline sleep conditions. The orange violin plots represent the biopsychological response of the young and old groups under sleep deprivation conditions; ***p < 0.001.

There was a significant interaction effect on the biopsychological response (Figure 1). No significant change was observed in older adults between SA and SD (*P* > 0.05), whereas young adults exhibited an increased biopsychological response in SD compared to SA (*P* < 0.001), as well as higher biopsychological response in SD compared to older adults under the same condition (*P* < 0.001).

### 3.2 Structural gray matter changes after partial SD

**FIGURE 2:**
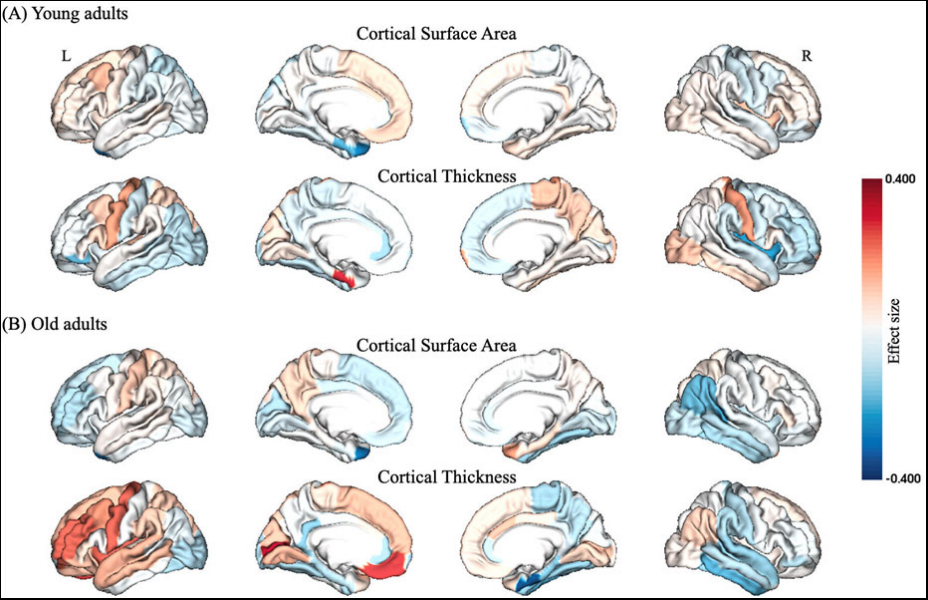
Structural gray matter changes after partial SD in **(A)** young adults and **(B)** old adults, respectively. The color bar of brain regions represented effect size between groups. L, left; R, right.

Young adults exhibited decreased CSA of left caudal middle frontal cortex and CT of EC, but increased CSA of left temporal pole and CT of right insula after partial SD (Figure 2A and Table 2). However, old adults showed only increased CSA in widespread brain regions, including left superior frontal cortex, left temporal pole, as well as increased CT of left isthmus cingulate cortex, right EC and para hippocampal cortex (Figure 2B and Table 3).

**TABLE 2:** Comparison of cortical surface area and thickness between groups in young adults.

Region	Measurement type	YgSA.mean	YgSA.sd	YgSD.mean	YgSD.sd	Ttest.P	Ttest.T	Ttest.EffectSize
Left caudal middle frontal	Area	2,614.19	447.88	2,557.26	422.48	0.0380	2.1425	0.1308
Left entorhinal	Area	420.14	97.52	438.42	78.04	0.0377	−0.1463	−0.207
Left temporal pole	Area	529.58	64.63	548.14	65.97	0.0252	−0.3216	−0.2842
Left entorhinal	Thickness	3.17	0.28	3.08	0.33	0.0261	2.3064	0.2898
Right insula	Thickness	3.01	0.13	3.04	0.13	0.0389	−0.1316	−0.2424

Region: brain region; measurement type: area (surface area) or thickness. YgSA: young group sleep adequate; YgSD: young group sleep deprivation. YgSA.mean/YgSA.sd: mean and standard deviation for the young group under sleep adequate conditions. YgSD.mean/YgSD.sd: mean and standard deviation for the young group under sleep deprivation conditions. Ttest.P/Ttest.T: *P*-value and T-value from the independent samples *t*-test. Ttest.EffectSize: effect size (Cohen’s d).

**TABLE 3:** Comparison of cortical surface area and thickness between groups in older adults.

Region	Measurement type	OdSA.mean	OdSA.sd	OdSD.mean	OdSD.sd	Ttest.P	Ttest.T	Ttest.EffectSize
Left cuneus	Area	1,475.45	193.08	1,498.34	197.99	0.0169	−2.5029	−0.1171
Left lateral occipital	Area	4,960.45	656.54	5,012.05	638.11	0.0126	−2.6224	−0.0797
Left superior frontal	Area	7,458.45	1,004.63	7,555.11	1,001.86	0.0417	−2.1095	−0.0963
Left temporal pole	Area	505.37	88.49	531.79	85.54	0.0211	−2.4091	−0.3036
Right bankssts	Area	794.38	136.24	824.39	139.59	0.0225	−2.3807	−0.2210
Left isthmus cingulate	Thickness	2.18	0.15	2.20	0.16	0.0477	−2.0478	−0.1556
Left pericalcarine	Thickness	1.81	0.16	1.76	0.15	0.0014	3.4425	0.3115
Right entorhinal	Thickness	3.17	0.38	3.30	0.33	0.0394	−2.1351	−0.3675
Right para hippocampal	Thickness	2.64	0.19	2.67	0.19	0.0118	−2.6481	−0.1675

Region: brain region; measurement type: area (surface area) or thickness. OdSA, older group sleep adequate; OdSD, older group sleep deprivation. OdSA.mean/OdSA.sd: mean and standard deviation for the older group under sleep adequate conditions. OdSD.mean/OdSD.sd: mean and standard deviation for the older group under sleep deprivation conditions. Ttest.P/Ttest.T: *P*-value and T-value from the independent samples *t*-test. Ttest.EffectSize: effect size (Cohen’s d).

### 3.3 Structural gray matter changes associated with biopsychological response

**FIGURE 3:**
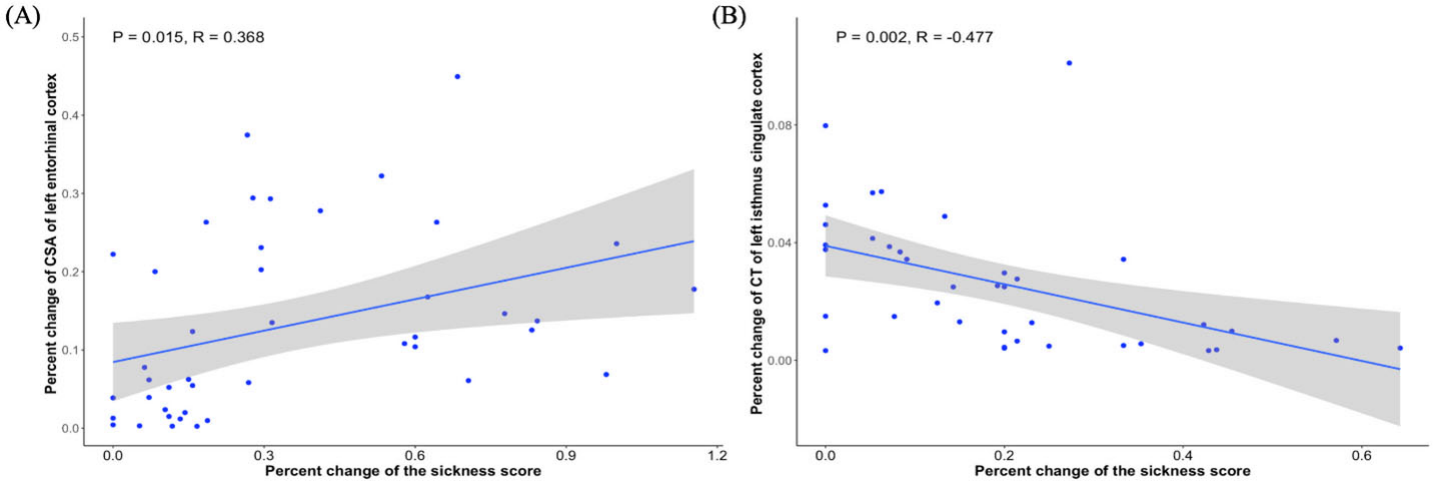
Relationship between gray matter change and biopsychological response in young adults and old adults. **(A)** Significant correlation between percent change in CSA of left EC and percent change in biopsychological response in young adults. **(B)** Significant correlation between percent change of CT of left isthmus cingulate cortex and percent change in biopsychological response in old adults.

A significant positive correlation was observed between the percent change in CSA of the left EC and the percent change in biopsychological response in young adults (*R* = 0.368, *P* = 0.015, Figure 3A), whereas older adults showed a significant negative correlation between the percent change in CT of the left isthmus cingulate cortex and the percent change in biopsychological response (*R* = −0.477, *P* = 0.002, Figure 3B).

## 4 Discussion

In this study, we have tested three hypotheses regarding age-related differences in gray matter responses to partial SD:

Firstly, we hypothesized that young adults exhibited reductions in CT and CSA predominantly in frontal regions. This hypothesis was partially supported: although reduced CSA in the left caudal middle frontal cortex and reduced CT in the EC were observed, unexpected increases in CSA (left temporal pole) and CT (right insula) were also present.

Secondly, we hypothesized that older adults would show increases in CT and CSA primarily in temporal regions. This hypothesis was only partially supported, as widespread structural increases were observed but not limited to the expected temporal areas; they included the left superior frontal cortex and left isthmus cingulate cortex.

Thirdly, we hypothesized that these structural changes would correlate significantly with biopsychological response, with differing directions of association across age groups. This hypothesis was fully supported: while young adults showed a positive correlation between changes in left EC CSA and biopsychological response, older adults exhibited a negative correlation between changes in left isthmus cingulate cortex CT and biopsychological response.

SD has been increasingly recognized as a physiological stressor that can induce mild systemic inflammation, characterized by elevated levels of pro-inflammatory cytokines such as interleukin-6 and C-reactive protein (Chennaoui et al., 2011; Everson, 2005; Irwin et al., 1996; Zielinski et al., 2014). This inflammatory response is not only relevant to immune function but also has potential implications for brain structure (Bucks et al., 2008; Jefferson et al., 2007). Preclinical and clinical studies have shown that inflammation can affect cortical morphology, including changes in CT and CSA, which are sensitive markers of neurodegenerative or neuroplastic processes (Gianaros et al., 2013; Satizabal et al., 2012). To assess the subjective experience of these physiological changes, the Sickness-Q questionnaire has been widely used to quantify individuals’ biopsychological responses to immune activation (Andreasson et al., 2018). Originally termed “sickness symptoms” or “sickness scores,” these measures reflect a constellation of fatigue, mood disturbance, cognitive impairment, and general malaise—collectively referred to as biopsychological response in this study (Dantzer et al., 2008). These established relationships provide a foundation for examining how age may modulate structural brain responses to partial sleep deprivation and their association with individual differences in biopsychological response.

For young adults, we found that following SD, there was a reduction in the CSA of the caudal middle frontal cortex (CMF). The prefrontal cortex is particularly sensitive to oxidative stress (Muzur et al., 2002), and SD notably affects neuropsychological performance associated with this region. The gray matter volume of the left caudal middle frontal cortex is positively correlated with spontaneous use of detailed encoding strategies (Husa et al., 2017). Therefore, the reduction in CSA of the CMF in young adults may be linked to diminished attention and decision-making abilities, further supporting the critical role of the prefrontal cortex in executive functions.

While for older adults, after partial SD, there was an increase in the CSA of the superior frontal gyrus (SFG). This change might represent an attempt by older adults to compensate for cognitive decline (Joo et al., 2013). Studies indicate that bilateral middle frontal gyri may support the spontaneous use of detailed encoding strategies by performing relational processing during encoding (Blumenfeld et al., 2011). Thus, the increase in CSA of the SFG in the elderly group may help maintain their cognitive function, particularly in the context of SD.

Besides, our findings showed that SD resulted in an increase in CSA but a decrease in CT of the left EC in young adults. The EC plays a crucial role in spatial learning, memory (Deng et al., 2009; Steffenach et al., 2005; Van Cauter et al., 2013) and general cognitive ability (Xu et al., 2024). Anatomically, it serves as an interface mediating communication between the hippocampus and neocortex (Canto et al., 2008; van Strien et al., 2009). Further analysis revealed a positive correlation between the percentage change in CSA of the left EC and the percentage change in biopsychological response, suggesting that structural alterations in this region may be linked to increased discomfort and functional impairment following SD.

For older adults, partial SD led to increased CT in the right EC, alongside a less pronounced increase in biopsychological response compared to younger adults. Neurotransmitter receptors in the EC are sensitive to SD, suggesting this increase may represent a compensatory mechanism for functional deficits, potentially involving changes in neuronal density or microstructure (Murata et al., 2018; Que et al., 2023). Studies indicate that prolonged sleep latency and reduced sleep efficiency correlate with decreased anterior-lateral EC volume, highlighting different adaptive mechanisms in older adults (Gervais et al., 2023). However, these adaptations do not fully mitigate the negative impacts of SD but rather relatively alleviate symptoms.

For young adults, SD resulted in increased CSA of the Temporal Pole (TP), which is vital for complex object and face recognition, visual processing, autobiographical memory, naming, word-object tagging, and semantic processing (Herlin et al., 2021). However, other studies have shown significant reductions in gray matter volume (GMV) and CT in the right TP following SD (Elvsåshagen et al., 2017). indicating potential cognitive and emotional regulation issues. In older adults, similar increases in CSA were observed in both the left TP and the right Banks of the Superior Temporal Sulcus (Bankssts). Poor sleep quality has been linked to cortical thinning in the Superior Temporal Sulcus (STS), with STS thickness mediating the relationship between emotional symptoms and sleep quality (Wang et al., 2021), These structural changes, particularly in the STS, may underlie the neural basis linking anxiety and depressive symptoms with poor sleep quality. The increase in CSA of these regions in older adults likely serves as a compensatory mechanism to mitigate functional impairments due to SD. Whether these adaptations are beneficial long-term remains unclear, especially given the incomplete understanding of underlying mechanisms in older adults. Results found that the elderly group exhibited an increase in the CSA of the left cuneus, lateral occipital cortex (LOC), and pericalcarine regions after SD. These areas play significant roles in visual processing and possess unique functional characteristics.

Following partial SD, young adults exhibited increased CT in the right insula, a region implicated in emotion regulation, interoceptive awareness, and stress response (Etkin and Wager, 2007). This finding aligns with previous reports of increased gray matter volume in the insula following acute SD and insomnia (Bushey et al., 2011; Diering, 2017; Tononi and Cirelli, 2014). Notably, such increases may reflect disrupted synaptic homeostasis, wherein SD interferes with the normal downscaling of synaptic strength during sleep, potentially leading to maladaptive structural changes (Tononi and Cirelli, 2014). Importantly, increased insular CT has been associated with heightened anxiety and impaired emotional regulation (Du et al., 2023), suggesting that the observed structural alterations may reflect adverse effects of SD on affective processing. These findings highlight the insula as a key region vulnerable to short-term sleep loss, with potential implications for mental health.

Following SD, the elderly group exhibited increased CT in the left isthmus of the cingulate, with a negative correlation to disease score changes. Despite previous findings of lower CT in insomniacs’ cingulate cortex (Grau-Rivera et al., 2020; Yan et al., 2018). our results suggest an increase in CT post-SD, likely due to disrupted synaptic pruning, resulting in greater synaptic strength and expanded gray matter density (Bushey et al., 2011; Diering, 2017; Tononi and Cirelli, 2014). Moreover, the significant negative correlation between increased CT in the isthmus of the cingulate and lower percentage changes in disease scores suggests this structural change may have some positive aspects. While the increase in CT might reflect a coping mechanism for SD, it could also relate to emotion regulation and cognitive function. Higher depression scores are linked to smaller volumes in the isthmus of the cingulate, and higher somatic symptom scores correlate with smaller volumes in the posterior cingulate cortex (PCC) (McLaren et al., 2016). Therefore, increased CT in the left isthmus of the cingulate may help mitigate adverse emotional and cognitive effects of SD in the elderly.

However, poor sleep quality leads to increased tissue volume loss in the hippocampus and PCC, key regions for cognitive decline and dementia (Sehar et al., 2024). This highlights the need to investigate whether the observed increase in CT in the isthmus of the cingulate is beneficial long-term and how it affects cognitive and emotional health in the elderly. In summary, the increase in CT in the left isthmus of the cingulate following SD may be a consequence rather than an adaptive response.

Our analysis revealed age-specific patterns of structural brain changes following partial SD. In young adults, alterations were observed in the left prefrontal cortex, left temporal pole, and right insula, regions associated with cognitive and emotional processing. In older adults, changes were found in the left EC, right temporal pole, left cuneus, lateral occipital cortex, pericalcarine, and left isthmus of the cingulate. These findings suggest that SD may affect distinct neural circuits in a manner that varies across the lifespan. While the results contribute to our understanding of how sleep loss impacts brain structure, further research is needed to explore their implications for insomnia, brain plasticity, and public health.

In this study, several limitations should be acknowledged. First, the sample size is relatively small with limited statistical power and generalizability of the results. Future studies should consider increasing the sample size to ensure the robustness of the findings. Second, although the immediate effects of SD were examined, the long-term changes in brain structure and function remain unclear. Third, we did not analyze potential gender differences due to limited statistical power when further dividing participants by both age and gender—particularly in the older adult group. Longitudinal studies would help to reveal the sustained effects of SD on the brain and its functions over time.

## 5 Conclusion

In summary, partial SD had more pronounced effects on brain structure in young adults compared to older adults. This may reflect age-related differences in inflammatory responses, with younger individuals showing stronger reactions linked to greater biopsychological symptoms and structural changes. Older adults, in contrast, may benefit from immune adaptations or compensatory mechanisms that reduce SD’s impact. These findings suggest differential vulnerability to sleep loss across the lifespan and highlight the need for further research on underlying mechanisms.

## Data Availability

The original contributions presented in this study are included in this article/supplementary material, further inquiries can be directed to the corresponding authors.

## Footnotes

1
https://openneuro.org/datasets/ds000201

2
http://surfer.nmr.mgh.harvard.edu

3
https://openfmri.org/dataset/ds000201
